# Robustness of Deep Learning Algorithm to Varying Imaging Conditions in Detecting Low Contrast Objects in Computed Tomography Phantom Images: In Comparison to 12 Radiologists

**DOI:** 10.3390/diagnostics11030410

**Published:** 2021-02-28

**Authors:** Hae Young Kim, Kyeorye Lee, Won Chang, Youngjune Kim, Sungsoo Lee, Dong Yul Oh, Leonard Sunwoo, Yoon Jin Lee, Young Hoon Kim

**Affiliations:** 1 Department of Radiology, Seoul National University Bundang Hospital, Seongnam-si, Gyeonggi-do 13620, Korea; qkfmrp860329@gmail.com (H.Y.K.); kyjsc0626@gmail.com (Y.K.); leonard.sunwoo@gmail.com (L.S.); yoonjin319@gmail.com (Y.J.L.); yhkrad@gmail.com (Y.H.K.); 2 Interdisciplinary Program in Bioengineering, Seoul National University, Seoul 08826, Korea; 92leekr@naver.com (K.L.); dyoh1003@gmail.com (D.Y.O.); 3 PROMEDIS, Seocho-gu, Seoul 06714, Korea; lss0328@gmail.com

**Keywords:** deep learning, tomography, X-ray computed, phantoms, imaging, artificial intelligence

## Abstract

The performance of deep learning algorithm (DLA) to that of radiologists was compared in detecting low contrast objects in CT phantom images under various imaging conditions. For training, 10,000 images were created using American College of Radiology CT phantom as the background. In half of the images, objects of 3–20 mm size and 5–30 HU contrast difference were generated in random locations. Binary responses were used as the ground truth. For testing, 640 images of Catphan^®^ phantom were used, half of which had objects of either 5 or 9 mm size with 10 HU contrast difference. Twelve radiologists evaluated the presence of objects on a five-point scale. The performances of the DLA and radiologists were compared across different imaging conditions in terms of area under receiver operating characteristics curve (AUC). Multi-reader multi-case AUC and Hanley and McNeil tests were used. We performed post-hoc analysis using bootstrapping and verified that the DLA is less affected by the changing imaging conditions. The AUC of DLA was consistently higher than those of the radiologists across different imaging conditions (*p* < 0.0001), and it was less affected by varying imaging conditions. The DLA outperformed the radiologists and showed more robust performance under varying imaging conditions.

## 1. Introduction

The increasing role of imaging in diagnostic processes, along with technological advances facilitating access to imaging, has resulted in an unprecedented amount of clinical workload for radiologists [1,2]. This has led to increasing interest in the medical society in developing techniques for automated imaging analysis that may improve the efficiency of radiological diagnosis [3]. Deep learning (DL) based on a convolutional neural network (CNN) has particularly gained attention from both the research community and start-up endeavors as a state-of-the-art technique for computer vision tasks such as automated imaging analyses [4,5].

An area of active research in DL-based imaging analysis has been the development of techniques for object detection on computed tomography (CT). Although these techniques have shown promising performance in previous studies [6,7,8,9], more research should be conducted on validating their robustness before they could be utilized in daily clinical practice. Existing studies have largely focused on high-contrast objects (ones that have a considerable attenuation difference with the background), such as lung nodules or calcifications in mammography. Clinical practice, particularly when it comes to the abdomen and pelvis, involves detection of low-contrast objects such as pancreatic cancer or metastasis in the liver [10]. While it is well known that performance of radiologists is affected substantially by changes in imaging conditions, such as radiation dose, object size, or the reconstruction algorithm used [11,12,13,14], more research is demanded regarding whether and to what degree the performance of DL techniques is affected by such variations in imaging conditions.

To this end, we used images of a CT phantom acquired under varying radiation dose settings, reconstruction algorithms, and object sizes to measure and compare the performance of a deep learning algorithm (DLA) with that of 12 radiologists in the detection of low-contrast objects across various imaging conditions. Using the CT phantom images, we could isolate the influence of varying imaging conditions of interest while controlling the other factors. 

## 2. Materials and Methods

No IRB approval was required for this phantom study. The data regarding the performance of the 12 radiologists were obtained from a previous study [15], which were aimed at comparing images denoised by a DLA with those reconstructed using advanced modeled iterative reconstruction (ADMIRE) and filtered back projection (FBP), in terms of the physical properties and radiologist performance in object detection. We used a DLA based on the deep residual learning framework [16]. The model consists of seven levels of residual blocks. Batch normalization layer follows each convolution layer in the residual block for stable training and is activated by the rectified linear unit. The number of convolution filters in each block is 64, 128, 256, 512, 1024, 2048, and 4096. For each level, max-pooling operation that reduces the input size in half is attached at the residual block. A fully-connected layer is applied to the end of the model and is activated by the softmax function that produces the probabilities of the presence of an object. The model is trained from scratch with initial learning rate of 0.0001. Our model architecture can be found at https://github.com/siniphia/PhantomDetectability (accessed on 27 February 2021).

### 2.1. CT Phantom and Protocol for Training Set

**Figure 1:**
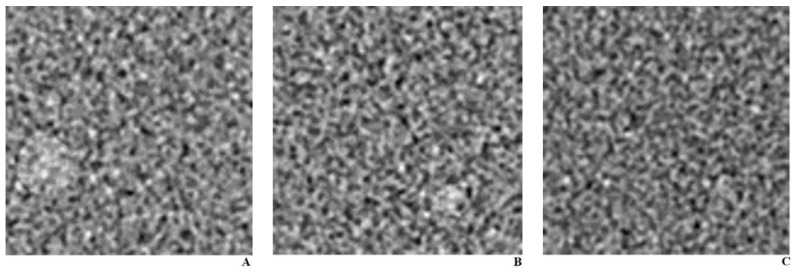
Representative images for the training set. CT phantom images with an artificially generated object of (**A**). 12 mm size at the left lower quadrant with a 10 HU difference to the background, and (**B**). 7 mm size object at the right lower quadrant with a 10 HU difference to the background, and (**C**). an image without any object.

For training the DLA, we used the CT image of American College of Radiology (ACR) CT accreditation phantom (model 464, Gammex–RMI) acquired under 100 kVp and 200 mAs and reconstructed using the FBP. We used a single CT machine (SOMATOM Definition Edge, Siemens Healthcare, Erlangen, Germany) (Table 1). For the generalizability of our DLA, it seemed practical that the algorithm be trained with images that could be acquired easily. Thus, we selected the radiation dose and reconstruction algorithm that are most prevalently used in daily practice. Moreover, we used a single fixed imaging condition for the training, to prove that our DLA can also perform well for testing set images acquired under different imaging conditions. We cropped the image to a size of 5 × 5 cm² and used it as a homogeneous background, and then artificially generated objects of varying size (ranging from 3 to 20 mm) by increasing the pixel values (ranging from 5 to 30 HU) at random locations (Figure 1). We created 10,000 images in total, half of which had a single object present, while the remaining half did not have any object. We fed the binary response (object present or absent) as the ground truth.

### 2.2. CT Phantom and Protocol for Testing Set

**Figure 2:**
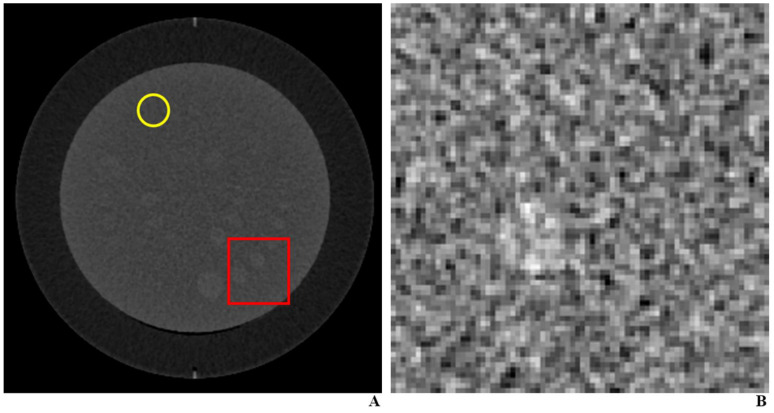
Representative images for the testing set. (**A**). We cropped the images of CT phantom to 5 × 5 cm² size (red box) so that an object of 9-mm or 5-mm size with +10 Hounsfield unit difference would either be absent or present in random locations. We hid unnecessary objects by covering them with object-absent image patches (i.e., a patch of the background as indicated by the yellow circle), leaving only a single object of choice. (**B**). The final image.

For the testing of the DLA, we used CT images of Catphan^®^ low-contrast phantom module (CTP 515) acquired under various doses (100 kVp; 200, 100, 50, 26 mAs) using a single CT machine (SOMATOM Definition Edge, Siemens Healthcare) (Table 1). We cropped the images to a size of 5 × 5 cm² so that an object would either be absent or present at random locations rather than just at the center (Figure 2). We used supra-slice objects of either 9- or 5-mm size with +10 Hounsfield unit difference with respect to the background. Leaving only a single object of choice, we hid other unnecessary objects by covering them with object-absent image patches. We reconstructed all the images using both ADMIRE and FBP. As mentioned previously, these images were originally created for a previous study [15]. We tested a total of 640 images (40 images (20 images with and 20 images without an object) × 2 reconstruction algorithms (i.e., ADMIRE and FBP) × 4 different radiation doses × 2 different object sizes).

### 2.3. Performance of the DLA in the Testing Set

**Figure 3:**
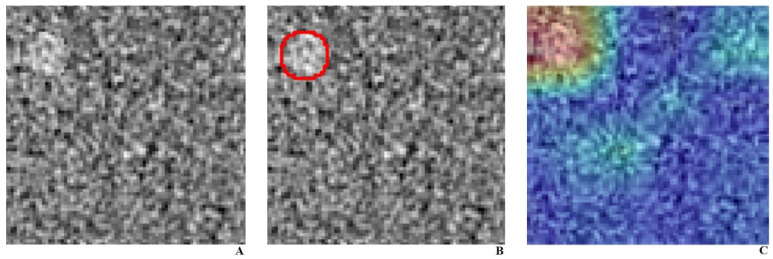
CT phantom image with a 9 mm object at the left upper quadrant, with a superimposed heatmap using gradient-weighted class activation mapping. (**A**). CT phantom image acquired under 100 kVp, 200 mAs, reconstructed with ADMIRE, with a 9 mm object, as (**B**). localized in a red circle. (**C**). Corresponding class activation map highlighting the activation in the left upper quadrant.

The DLA produced probabilities of binary classes (0 for absence and 1 for presence) per each object using the softmax function. We acquired heat maps (Figure 3) using gradient-weighted class activation mapping (Grad-CAM), which is a class-discriminative localization technique that can render visual explanations to make CNN-based models more interpretable [17].

### 2.4. Performance of Radiologists in the Testing Set

To test the performance of radiologists, we used the graphical user interface (GUI) using a Python programming toolkit (Tkinter), where we set the default display to a window level of 70, which was the mean Hounsfield unit value of the image background, and a window width of 100 [15]. We numbered the images in random order and displayed the even-numbered images on the left side of the screen, and odd-numbered images on the right side of the screen. This was to minimize bias caused by change detection in the flicker paradigm [18,19,20].

Twelve radiologists with varying degree of experience (six attending radiologists from three different institutions with 6–24 years of clinical experience each, and six radiologists in training from a single institution) evaluated the presence or absence of objects on a five-point confidence scale (1: definitely absent, 2: probably absent, 3: indeterminate, 4: probably present, and 5: definitely present). Before the review, the radiologists underwent two sessions of tutorials, each of which consisted of ten questions and instant feedback on correct answers. After the tutorial, each radiologist independently reviewed the 960 images: 40 images (half of which had an object present) × 3 reconstruction methods (FBP, ADMIRE, DL-based denoising algorithm) × 4 radiation doses (100 kVp; 200, 100, 50, 26 mAs) × 2 object sizes (5 and 9 mm). As this image review was originally intended for a previous study, where we tested the performance of a DL-based denoising algorithm, we only used 640 images out of the 960 images (excluding the images reconstructed with the DL-based denoising algorithm) for the current study. We asked the radiologists to use a display calibrated to the DICOM standard and minimize reading room light as much as possible.

### 2.5. Performance Using Classic Computer Vision Approach–Template Matching

We focused on deep learning algorithm instead of a classic computer vision approach in this study. As deep learning frameworks can be re-trained using a custom dataset for other uses, deep learning renders more flexibility in diverse applications compared to classic computer vision algorithms that tend to more domain-specific. Nevertheless, classic computer vision approach is not obsolete, and there are cases where such approaches are more efficient while simpler than deep learning algorithms. Thus, we also applied a computer vision algorithm to our dataset. We specifically used the template matching method, which uses a moving template image to scan the target image, calculates similarity scores per step, and finds the most similar object compared with the template.

### 2.6. Statistical Analysis

The sample size was determined from a previous study [15], with the aim of proving noninferiority of the DL-based denoising algorithm to ADMIRE in low-contrast object detection.

We measured and compared the area under the ROC curve (AUC) of DLA and 12 radiologists, first irrespective of the imaging conditions, and then across various imaging conditions. We used the multi-reader multi-case (MRMC) AUC to pool the data of the radiologists. We conducted the Hanley and McNeil test for [21] the comparisons of the AUCs. We corrected the familywise type-I error via Benjamini & Hochberg correction and considered a *p*-value < 0.00625 as statistically significant.

For our secondary analysis by object sizes, we repeated the comparisons as described above, separately for 9 mm and 5 mm objects. Low-contrast object detection is clinically more relevant for objects of at least 9 mm size than for objects as small as 5 mm.

Based on our secondary analysis, we noted that the performance of the DLA in detecting the 9 mm objects seemed more stable across various imaging conditions in comparison with that of the 12 radiologists. To statistically prove our hypothesis that the DLA is relatively more robust to varying imaging conditions than the radiologists, we performed *post-hoc* analyses as follows. We compared the performances of the DLA and radiologists in terms of the (1) reduction in the AUC across radiation doses (e.g., reduction in the AUC from 200 mAs to 26 mAs, or that from 200 mAs to 50 mAs), and (2) reduction in the AUC across ADMIRE and FBP. For the comparisons, we performed bootstrapping of 2000 replications with replacement. For example, we made 2000 measurements for the reduction in the AUC from 200 mAs to 26 mAs, for both DLA and for the 12 radiologists. As the measurements followed a standard normal distribution, we used the z-tests to compare the AUC reduction between the DLA and the 12 radiologists. We did not perform the same analysis for the 5 mm objects, as the AUCs of the 12 radiologists were mostly below 0.6 across all the imaging conditions, and therefore, the apparent stability of the AUCs across the imaging conditions did not bear any clinical significance. We corrected the familywise type-I error via Benjamini & Hochberg correction and considered a *p*-value < 0.0125 as statistically significant.

To calculate the AUC in detecting the 9 mm and 5 mm objects via template matching method, we applied ten thresholds having fixed intervals from 0 to 1.

We performed all the statistical analyses using iMRMC, software version 4.0.0 (Division of Imaging, Diagnostics, and Software Reliability, OSEL/CDRH/FDA) and R, version 3.5.2 (The R Foundation for Statistical Computing).

## 3. Results

### 3.1. Primary Analysis

**Figure 4:**
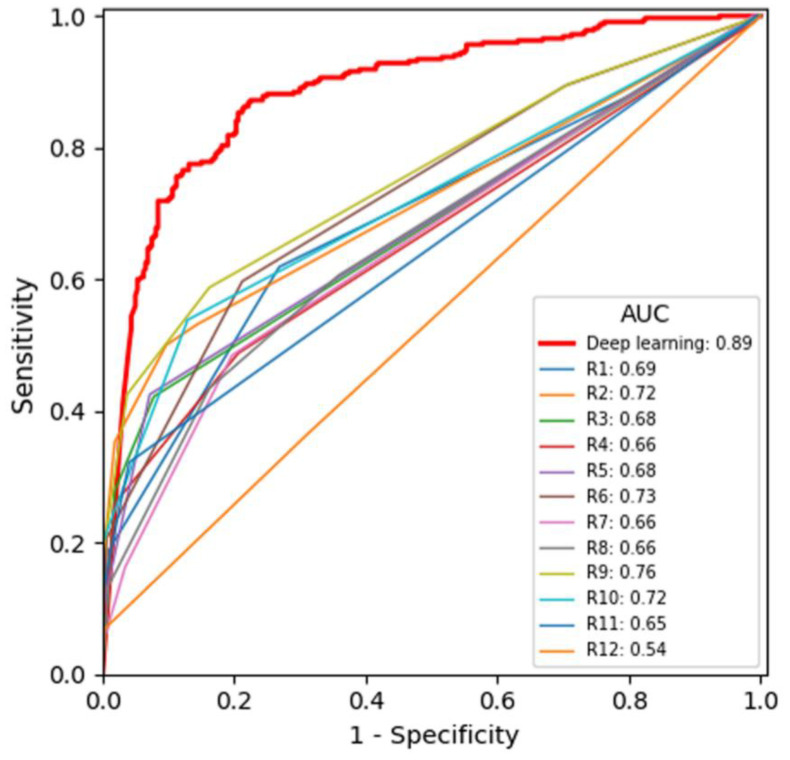
Receiver operating characteristic curves of the deep learning algorithm and the 12 radiologists. Radiologists R7–R12 are residents, whereas R1–R6 are attending radiologists. AUC = area under the curve.

The AUC of the DLA was significantly higher than that of the 12 radiologists (0.886 vs. 0.678; difference, 0.208 (95% CI, 0.205–0.213); *p*-value < 0.0001) (Table 2, Figure 4). The AUC of the DLA was consistently and significantly higher than that of the 12 radiologists across different radiation doses, reconstruction methods, and object sizes (*p* values were all less than 0.0001) (Table 2).

### 3.2. Secondary Analysis by Object Size

The AUC of the DLA was significantly higher than that of the 12 radiologists in the detection of both 9 mm objects (0.979 vs. 0.776; difference, 0.203, 95% CI, 0.159–0.247; *p*-value < 0.0001) and 5 mm objects (0.763 vs. 0.581; difference, 0.182, 95% CI, 0.179–0.185; *p*-value < 0.0001) (Table 3). The superior AUC of the DLA was consistently observed across the different imaging conditions, for both 5 mm and 9 mm object sizes. In the detection of 9 mm objects, the AUC of the DLA was 0.945 or higher under all the imaging conditions (Table 3). In the detection of 5 mm objects, the AUC of the DLA was 0.956 under the 200 mAs dose but 0.658 under the 26 mAs dose. In the detection of 5 mm objects, all the AUCs of the 12 radiologists were 0.599 or below, except when the radiation dose was 200 mAs (Table 3).

### 3.3. Post-Hoc Analysis

The reduction in the AUC across radiation doses and reconstruction methods was significantly lower for the DLA compared with that of the radiologists (*p* values were all less than 0.0001), indicating that the performance of the DLA was relatively more robust to the changes in the imaging conditions than that of the radiologists (Table 4).

### 3.4. Template Matching Method

The AUCs in detecting 5 mm target and 9 mm target by applying the template matching method were generally lower compared to the AUCs using the DLA (Supplementary Table S1). AUC was 0.69 and 0.65 for 5 mm and 9 mm target, respectively.

## 4. Discussion

The DLA outperformed the 12 radiologists in detecting low-contrast objects, consistently across various imaging conditions such as the radiation dose, object size, and reconstruction method used. In the detection of the 9 mm objects, the DLA showed an AUC of over 0.9 under all the conditions, even for the lowest radiation dose tested (100 kVp, 26 mAs). The performance of the DLA was relatively more robust to the changes in the imaging conditions than that of the 12 radiologists, showing a significantly lower degree of AUC reduction when the radiation dose or reconstruction algorithm was altered. Our results show potential for the clinical application of DL algorithms to low-dose CT protocols for screening or surveillance purposes.

Our study has the following strengths. First, we fill the existing knowledge gap in literature by confirming that DL algorithms can indeed be robust to changing imaging conditions when detecting low-contrast objects. While it is known that the performance of radiologists is affected significantly by the imaging conditions, such as radiation dose or reconstruction algorithm used [11,12,13,14], studies on such performance variations for DL algorithms have been lacking. A previous study [22] that used CT phantom images to investigate how imaging conditions such as object size, radiation dose, slice thickness, or reconstruction methods affect the performance of a DLA in detecting pulmonary nodules reported the performance of the algorithm itself, but not in comparison to that of radiologists. Moreover, the prior study tested high contrast object detection using pulmonary nodules, while we focused on low contrast object detection, which is more relevant when it comes to object detection in abdominal and pelvic organs. To the best of our knowledge, this is the first study that compares the performance of a DLA with that of radiologists in low-contrast object detection across varying imaging conditions. Another strength of this study is that we tested more than 600 images, involving 12 radiologists. Our receiver operating characteristic curves of the DLA and the radiologists show that the DLA achieved significantly superior performance, even in comparison to the attending radiologists. Finally, by using a CT phantom, we could control the imaging factors other than the one we were interested in.

However, some of the limitations of our study are as follows. First, although we could control the imaging factors strictly using CT phantom images, it raises a serious concern on whether our results can be reproduced in CT images of the actual human body. The task in our study was very simple: object detection in a homogeneous background without any variation. In contrast, object detection in an actual human body is more complex, as normal anatomical structures can be misinterpreted (i.e., causing false-positives) as target objects. Second, we only tested hyperattenuating objects. In actual clinical practice, lesions of interest may be hypoattenuating compared with the background (e.g., hepatic metastasis from pancreatic cancer). Third, the images used for the training and testing sets were apparently very similar, raising concern of overfitting. Nonetheless, we tried to create differences by varying the object size and contrast difference with the background in the testing set. We fixed the imaging parameters, reconstruction algorithm (100 kVp, 200 mAs), and FBP for the training set images, and altered the conditions for the testing set images; even in this case, the DLA showed an excellent performance. Finally, we did not incorporate segmentation and object localization (x, y coordinates). To compensate for this, we used heat maps for the DLA and confirmed that the highest activation occurs at the targeted areas. However, we could not assess whether the radiologists had localized the objects correctly, as they only responded to the presence/absence of an object on a five-point Likert scale.

In summary, our DLA outperformed 12 radiologists in detecting low-contrast objects across various imaging conditions. The performance of DLA was also relatively more robust (i.e., stable) to changes in the imaging conditions than that of the 12 radiologists.

## Figures and Tables

**Table 1:** Computed tomography (CT) scan and reconstruction parameters for the training and testing of the DLA.

Parameter	Data
Reference Tube Current-Time Product of Training set	200
Reference Tube Current-Time Product of Testing set	200, 100, 50, 26
Tube potential (kVp)	100
Collimation (mm)	128 × 0.6
Section thickness (mm)	4
Rotation time (s)	0.5
Pitch (mm)	0.6
Scan field of view (cm)	60
Display field of view (cm)	50
Reconstruction in Training set	FBP
Reconstruction in Testing set	Both ADMIRE-3, FBP
Kernel for FBP	I40f, B40f

DLA = deep learning algorithm, FBP = filtered back projection, ADMIRE = advanced modeled iterative reconstruction.

**Table 2:** Primary analysis.

-	DLA	Radiologists	Difference (95% CI)	*p*-Value
Total	0.886 (0.859–0.912)	0.678 (0.638–0.717)	0.208 (0.205–0.213)	<0.0001
Radiation Dose				
26 mAs	0.825 (0.759–0.890)	0.570 (0.526–0.614)	0.255 (0.248–0.264)	<0.0001
50 mAs	0.789 (0.719–0.859)	0.613 (0.561–0.664)	0.176 (0.168–0.183)	<0.0001
100 mAs	0.926 (0.882–0.970)	0.725 (0.671–0.778)	0.201 (0.188–0.211)	<0.0001
200 mAs	0.982 (0.966–0.998)	0.812 (0.760–0.864)	0.170 (0.166–0.180)	<0.0001
Reconstruction				
FBP	0.885 (0.849–0.921)	0.653 (0.611–0.695)	0.232 (0.227–0.241)	<0.0001
ADMIRE	0.904 (0.870–0.904)	0.705 (0.658–0.752)	0.199 (0.195–0.204)	<0.0001
Object Size				
5 mm	0.763 (0.711–0.816)	0.581 (0.549–0.613)	0.182 (0.177–0.190)	<0.0001
9 mm	0.979 (0.965–0.993)	0.776 (0.720–0.833)	0.203 (0.196–0.211)	<0.0001

Data are the AUC with 95% CIs in parentheses. *p*-value < 0.00625 was considered as statistically significant. AUC = area under receiver operating characteristics curve, CI = confidence interval, DLA = deep learning algorithm, FBP = filtered back projection, ADMIRE = advanced modeled iterative reconstruction.

**Table 3:** Secondary analysis by object size.

-	DLA	Radiologists	Difference (95% CI)	*p*-Value
**For Detection of 9 mm Objects**				
Total	0.979 (0.965–0.993)	0.776 (0.720–0.833)	0.203 (0.159–0.247)	<0.0001
Radiation Dose				
26 mAs	0.965 (0.930–1.000)	0.602 (0.535–0.668)	0.363 (0.359–0.367)	<0.0001
50 mAs	0.945 (0.890–1.000)	0.679 (0.594–0.764)	0.266 (0.260–0.272)	<0.0001
100 mAs	0.999 (0.996–1.000)	0.866 (0.797–0.936)	0.133 (0.131–0.135)	<0.0001
200 mAs	0.998 (0.992–1.000)	0.964 (0.941–0.988)	0.034 (0.033–0.035)	<0.0001
Reconstruction				
FBP	0.980 (0.964–0.997)	0.744 (0.683–0.805)	0.236 (0.234–0.238)	<0.0001
ADMIRE	0.986 (0.968–1.000)	0.813 (0.749–0.877)	0.173 (0.171–0.175)	<0.0001
**For Detection of 5 mm Objects**				
Total	0.763 (0.711–0.816)	0.581 (0.549–0.613)	0.182 (0.179–0.185)	<0.0001
Radiation Dose				
26 mAs	0.658 (0.534–0.781)	0.541 (0.498–0.584)	0.117 (0.105–0.129)	<0.0001
50 mAs	0.615 (0.491–1.738)	0.549 (0.500–0.598)	0.066 (0.054–0.078)	<0.0001
100 mAs	0.820 (0.722–0.917)	0.577 (0.531–0.623)	0.243 (0.233–0.253)	<0.0001
200 mAs	0.956 (0.916–0.996)	0.661 (0.593–0.729)	0.295 (0.288–0.302)	<0.0001
Reconstruction				
FBP	0.763 (0.689–0.837)	0.564 (0.534–0.595)	0.199 (0.197–0.201)	<0.0001
ADMIRE	0.785 (0.714–0.855)	0.599 (0.554–0.643)	0.186 (0.183–0.189)	<0.0001

Data are the AUC with 95% CIs in parentheses. *p*-value < 0.0083 was considered as statistically significant. AUC = area under receiver operating characteristics curve, CI = confidence interval, DLA = deep learning algorithm, FBP = filtered back projection, ADMIRE = advanced modeled iterative reconstruction.

**Table 4:** Comparison of AUC reductions across imaging conditions for DLA and 12 radiologists in detecting 9 mm objects.

AUC Reduction	DLA	Radiologists	Difference (95% CI)	*p*-Value
Between 200 mAs and 26 mAs	0.032	0.362	−0.330 (−0.366 to −0.294)	<0.0001
Between 200 mAs and 50 mAs	0.052	0.284	−0.233 (−0.290 to −0.170)	<0.0001
Between 200 mAs and 100 mAs	0.003	0.98	−0.095 (−0.112 to −0.078)	<0.0001
Between ADMIRE and FBP	0.011	0.069	−0.058 (−0.09 to −0.034)	<0.0001

Data are the AUC with 95% CIs in parentheses. *p*-value < 0.0125 was considered as statistically significant. AUC = area under receiver operating characteristics curve, CI = confidence interval, DLA = deep learning algorithm, FBP = filtered back projection, ADMIRE = advanced modeled iterative reconstruction.

## Data Availability

Data sharing not applicable.

## Supplementary Materials

The following are available online at https://www.mdpi.com/2075-4418/11/3/410/s1, Table S1: title: Object Detection Using Template Matching Method.

## Abbreviations

ADMIRE	Advanced modeled iterative reconstruction
CNN	convolutional neural network
DL	Deep learning
DLA	Deep learning-based algorithm
FBP	Filtered back projection
GUI	Graphical user interface
MRMC	Multi-reader multi-case
