# Supplementary material for: Robustness of Deep Learning Algorithm to Varying Imaging Conditions in Detecting Low Contrast Objects in Computed Tomography Phantom Images: In Comparison to 12 Radiologists
Source: Diagnostics (Basel). 2021 Feb 28;11(3):410. doi: 10.3390/diagnostics11030410 (PMC7997324; doi:10.3390/diagnostics11030410)
Supplement: Supplementary file 1 [file diagnostics-11-00410-s001.pdf]

Supplementary Table S1. Object Detection Using Template Matching Method.

	Template Matching (5 mm)	Template Matching (9 mm)
**Total**	0.731 (0.692–0.769)	0.669 (0.627–0.710)
**Radiation Dose**		
26 mAs	0.712 (0.633–0.792)	0.681 (0.596–0.765)
50 mAs	0.655 (0.570–0.740)	0.616 (0.528–0.703)
100 mAs	0.773 (0.702–0.845)	0.695 (0.613–0.777)
200 mAs	0.855 (0.798–0.912)	0.752 (0.678–0.828)
**Reconstruction**		
FBP	0.716 (0.660–0.772)	0.661 (0.601–0.721)
ADMIRE	0.757 (0.704–0.810)	0.688 (0.630–0.746)
**Object Size**		
5 mm	0.626 (0.565–0.687)	0.511 (0.447–0.574)
9 mm	0.836 (0.792–0.879)	0.822 (0.778–0.866)

Data are the AUC with 95% CIs in parentheses. AUC = area under receiver operating characteristics curve, CI = confidence interval, FBP = filtered back projection, ADMIRE = advanced modeled iterative reconstruction.
